# Metformin-Functionalized Selenium Nanoparticles Restore Insulin Signaling and Metabolic Homeostasis in Letrozole-Induced Polycystic Ovary Syndrome Rat Model

**DOI:** 10.7150/ijms.135170

**Published:** 2026-09-03

**Authors:** Uğur Ermis, Damla Binentoglu, Erdem Toktay, Huseyin Fatih Gul, Hasan Ilhan, Muhammed Yayla

**Affiliations:** 1 Department of Pharmacology; Faculty of Medicine; Kafkas University, 36100 Kars/Turkey.; 2 Department of Histology and Embryology; Faculty of Medicine; Kafkas University, 36100 Kars/Turkey.; 3 Department of Biochemistry; Faculty of Medicine; Kafkas University, 36100 Kars/Turkey.; 4 Department of Biotechnology; Biotechnology Institute; Ankara University, 06100 Ankara/Turkey.; 5 Department of Pharmacology; Faculty of Medicine; Selcuk University, 42100 Konya/Turkey.

**Keywords:** nanomedicine, insulin resistance, metformin, nanoselenium, rat

## Abstract

In this study, we designed a novel 'metformin-coated nanoselenium nanocomposite' (Met+SeNP) to leverage the synergistic potential of both agents delivered via a single nanoplatform, aiming to restore metabolic and hormonal homeostasis in a letrozole-induced PCOS rat model. Met+SeNP was synthesized via a redox reaction of sodium selenite and ascorbic acid in the presence of metformin, creating a stable metformin-functionalized selenium nanocomposite. The particles were characterized using UV-Vis spectroscopy, SEM, and EDX. Female Sprague-Dawley rats were divided into five groups: Healthy, PCOS (letrozole-induced), Metformin (300 mg/kg), SeNP (0.4 mg/kg), and Met+SeNP (nanocomposite dose, SeNP 0.4 mg/kg + Metformin 300 mg/kg). The nanocomposite treatment most effectively minimized weight gain (39.36% vs. PCOS controls), normalized HOMA-IR, and restored circulating LH, estrogen, and testosterone levels. Histopathological evaluation demonstrated a marked reduction in cystic follicles and recovery of corpus luteum formation following Met+SeNP therapy. Immunohistochemical analysis further revealed that Met+SeNP robustly reversed PCOS-induced suppression of INR, IRS-1, and IRS-2 expression in both liver and skeletal muscle tissues. In addition, the nanocomposite significantly improved serum ALT, AST, and ALP levels while maintaining normal urea and creatinine concentrations, supporting hepatic protection and systemic safety. The metformin-functionalized SeNP nanocomposite represents a promising potent therapeutic strategy for PCOS-associated metabolic dysfunction, outperforming monotherapies in restoring insulin signaling, endocrine balance, and tissue integrity.

## Introduction

Polycystic ovary syndrome (PCOS) is characterized by ovulation disorders, increased androgen levels and cysts on the ovaries. It affects 4-20% of women of reproductive age, making it one of the most common endocrinopathies 1. Alongside clinical symptoms such as hirsutism, menstrual irregularities and infertility, PCOS is also associated with cardiovascular risk factors such as insulin resistance (IR), dyslipidemia, chronic inflammation and endothelial dysfunction 2, 3. IR is present in around 80% of women with PCOS, contributing to the exacerbation of metabolic and reproductive disorders via hyperinsulinemia 4, 5. This increase not only brings with it the risk of infertility, but also long-term metabolic comorbidities such as type 2 diabetes and cardiovascular disease. Furthermore, the global economic burden of PCOS management is estimated to exceed billions of dollars annually, highlighting the urgent need for more effective, cost-efficient treatment strategies 6, 7.

Metformin is now widely used off-label in the treatment of PCOS. Metformin is an oral insulin sensitizer used in the treatment of type 2 diabetes, and it may be effective in reducing insulin resistance (IR), regulating the menstrual cycle, increasing ovulation and lowering serum androgen levels in PCOS 8, 9. However, high doses of metformin are often required to achieve therapeutic effects in PCOS patients, which frequently leads to lactic acidosis and gastrointestinal distress, thereby limiting patient compliance.

Selenium (Se) is an essential mineral with antioxidant and anti-inflammatory properties. It exerts its biological functions through selenoproteins and may reduce IR by lowering fasting blood sugar through an insulin-like effect 10. Elemental selenium in nanoparticulate form (SeNP) is notable for its low toxicity and high bioavailability 11; it holds therapeutic potential in diseases such as PCOS 12, diabetes 13, cancer 14 and Alzheimer's disease 15.

Although metformin and selenium are both effective when used individually, conventional oral administration of these substances presents challenges such as low bioavailability, rapid renal clearance and dose-dependent gastrointestinal side effects 16, 17. Nanotechnology offers a promising strategy to overcome these limitations. Specifically, reducing selenium to the nanoscale (SeNPs) significantly reduces its toxicity while enhancing its catalytic efficiency as an antioxidant compared to inorganic selenium forms 18, 19. Furthermore, surface modification, or 'coating', of nanoparticles is a critical approach in pharmaceutical design, improving stability, preventing aggregation and enabling targeted delivery 20. The aim of this study was therefore to design, synthesize and physiochemically characterize a novel metformin-coated nanoselenium nanocomposite, and evaluate its potential to restore metabolic and hormonal homeostasis in a letrozole-induced PCOS rat model.

In a previous study by Rabah et al., commercially available selenium nanoparticles (SeNPs) were administered alongside free metformin as two separate therapeutic agents. In contrast, this study involved developing a novel metformin-loaded selenium nanoparticle system through an in-situ synthesis approach, whereby metformin was incorporated during the formation of the nanoparticles. During reduction, metformin integrated into the developing selenium nanoparticle structure, forming a single nanoformulation rather than a physical mixture of free metformin and preformed SeNPs. This distinction is important because the in-situ incorporation of metformin is expected to provide a more sustained and controlled drug release profile than the rapid availability of free metformin in physical co-administration systems. Furthermore, nanoformulation may improve drug retention within the nanoparticle matrix, enhance formulation stability, and enable the simultaneous delivery of selenium and metformin from a single carrier platform. However, this potential advantage remains hypothetical, as no pharmacokinetic or in vitro drug release studies were conducted in this study. Similarly, the potential for improved drug retention, enhanced formulation stability and the simultaneous delivery of selenium and metformin from a single carrier platform requires further experimental confirmation. Additionally, while Rabah et al. focused on a PCOS model, this study evaluates the therapeutic potential of an in situ synthesized metformin-selenium nanoformulation for treating peripheral insulin resistance related to PCOS. To our knowledge, this is the first study to evaluate a metformin-coated selenium nanocomposite in a letrozole-induced PCOS model, while also examining insulin signaling proteins in liver and skeletal muscle tissues. It should be noted that the present study did not include a group that received free metformin and selenium nanoparticles directly. Therefore, the comparisons presented in this study are limited to the respective monotherapies, and no conclusions can be drawn regarding superiority over conventional combination treatment.

## Materials and Methods

### Chemicals

Metformin HCl (MEF12124334) was supplied as a pure active ingredient by ARIS Pharmaceuticals. Nanoparticulate drugs (SeNP and Met+SeNP) were synthesized by Assoc. Prof. Dr. Hasan İlhan at Ankara University, Biotechnology Institute. Letrozole (Letu™, Onko, Istanbul), xylazine (Xylasinbio™, Intermed, Ankara), and ketamine (Ketalar™, Pfizer, Istanbul) were purchased commercially.

### Processes for preparing metformin-coated nanoselenium

Met+SeNP was prepared using a redox reaction with sodium selenite and ascorbic acid according to a previously reported method 21. 7.5 g of metformin was dissolved in 100 ml of distilled water; 0.3 ml of 0.1 M Na₂SeO₃ and 2 ml of TW-80 (2 mg/ml) were added, and the mixture was kept at room temperature for 30 minutes. Then, a 0.1 M ascorbic acid solution was slowly added, and the mixture was left to react in the dark at 30 °C for 4 hours. Excess reagents were dialyzed against ultrapure water for 48 hours (MWCO 1000), then centrifuged at 15,000 rpm for 10 minutes. The resulting precipitate was collected, freeze-dried, and the final Met+SeNP product was obtained (Figure 1A).

### Characterisation

The synthesized Met+SeNP nanoparticles were characterized using various analytical techniques. UV-vis spectra were obtained using an Agilent/Cary 60 spectrophotometer, while morphology and surface analysis were performed using scanning electron microscopy (SEM) and energy-dispersive X-ray spectroscopy (EDX) (SU-1510, Hitachi, 200 kV). Infrared (IR) spectra were obtained using a Varianrxr/660 IR spectrometer in the 4000-400 cm-1 range, using KBr pellets (Figure 1B-E).

### Animals

The experiment used 25 female Sprague-Dawley rats weighing 170-190 g and aged 10-12 weeks. The study was conducted with the approval of the ethics committee KAÜ-HADYEK/2024-009. The animals were housed at 22±2 °C, 50-60% humidity, and a 12/12-hour light/dark cycle and were fed standard rat chow and water ad libitum.

### Experimental groups and drug administration

Group 1 Healthy: 0.5% oral Carboxymethyl Cellulose (CMC) 22.

Group 2 PCOS group: 1 mg/kg letrozole + 0.5% CMC, orally for 21 days 22, 23.

Group 3 PCOS+Metformin: Following 21 days, metformin at 300 mg/kg, orally for 15 days 23, 24.

Group 4 PCOS+SeNp: Following 21 days, SeNp at 0.4 mg/kg, orally, for 15 days 10, 25, 26

Group 5 PCOS+metformin-coated SeNP: Following 21 days, metformin-coated SeNP (0.4mg/kg SeNp+300 mg/kg metformin), orally for 15 days.

Weight monitoring was performed weekly in all experimental groups.

### Detection of polycystic ovary syndrome and insulin resistance

#### Vaginal cytology (smear) test

The estrus cycle in rats lasts 4-5 days, and vaginal cytology was performed during the last 5 days of letrozole administration. Cells stained with Giemsa stain were classified according to epithelial and neutrophil cells, and the formation of PCOS was confirmed by histological examination 23.

#### Oral glucose tolerance test

All rats were fasted overnight, and fasting blood glucose (FBG) was measured at 0 minutes, followed by administration of a 2 g/kg glucose solution, blood glucose measurements were performed at 30 and 120 minutes from the tail vein (On-Call Plus™, 50-500 mg/dl). The OGTT and serum insulin levels at the end of the experiment were used to calculate insulin resistance using the HOMA-IR formula [HOMA-IR = Insulin (µIU/ml) ×FBG (mg/dl)/405] 27.

### Surgical procedures, sample collection and analysis

Anesthesia was achieved by intraperitoneal administration of a combination of 75 mg/kg ketamine and 15 mg/kg xylazine. Blood samples were collected in biochemistry tubes and centrifuged at 2000 rpm for 10 min to obtain serum. Tissue samples from the ovaries, liver, left quadriceps muscle were carefully dissected. The ovaries were weighed and recorded, and all tissues were fixed in 3.7% formalin for 72 hours before being transferred to Eppendorf tubes and stored at -80 °C.

### Analysis of serums

Serum samples were analyzed for HDL, LDL, total cholesterol, triglycerides, FSH, LH, estradiol (E2), total testosterone, insulin, ALT, AST, ALP, urea and creatinine were measured using the ELISA Kits purchased from Mybiosource company, China. The analyses were performed in the Research and Development Laboratory of the Department of Medical Biochemistry, Faculty of Medicine, Kafkas University. Bio-Tek ELx50 automatic washer and Epoch™ Microplate Spectrophotometer (BioTek Instruments, USA) devices were used for the measurements.

### Analyses of tissues

At the end of the experiment, tissue samples were placed in a 3.7% formaldehyde solution and fixed for 72 hours. After fixation, we performed standard procedures as described previously. Finally, the tissues were held in a solution of molten paraffin (at 60°C) for 2 hours, and at the end of this process, the tissue samples were embedded in blocks suitable for sectioning 28.

### Hematoxylin and eosin staining process

Tissue blocks were placed in a microtome device, and 5-micrometer-thick sections were taken from each block onto polylysine-coated slides and kept in an oven at 65°C for 20 minutes. The nuclei and cytoplasm were stained for 5 minutes in Harris hematoxylin staining solution and for 2 minutes in Eosin Y solution. Finally, the tissue surfaces were covered with a lamella using mounting solution, and the tissue preparation process was completed as described previously 29.

### Immunohistochemical staining method

Sections taken from positively charged slides were soaked in distilled water for 5 minutes before undergoing immunohistochemical staining. Following incubation, primary antibodies (INR, IRS-1, and IRS-2, PAA895RA01, PAC546MU01, PAD880RA01, Cloud Clone®, China) were applied to the slide surface and incubated overnight at 4°C. 29. For quantitative analysis, five sections per rat were evaluated using ImageJ software (NIH, USA). The percentage of immunopositive area (% positive area) was calculated for each section, and group means were compared by statistical analysis 30.

### Microscopic examination and photography

The slides prepared after HE and IHC staining were examined and photographed using a computer-assisted Olympus BX43 microscope with a camera attachment. The photographs taken were combined using Adobe Photoshop CS5 software to facilitate comparative examination 31.

### Statistical analyses

Biochemical and histological data were analyzed using IBM SPSS Statistics 27.0.1 software. Numerical data were assessed for normal distribution using the Kolmogorov-Smirnov and Shapiro-Wilk tests; data following a normal distribution were compared using parametric tests (One-Way ANOVA) Tukey HSD was used for multiple comparisons when variances were homogeneous. Graphs were prepared using GraphPad Prism 9.3.0 software, and error bars were drawn according to standard deviation (SD). The significance level was accepted as p<0.05.

## Results

### Synthesis of nanoselenium and metformin-coated nanoselenium

SEM images show that Met+SeNP nanoparticles are generally spherical in shape, homogeneously distributed on the surface and have an average size of 150-180 nm. Following metformin loading, the structural integrity of the nanoparticles was maintained, and no significant agglomeration was observed, indicating their stability and suitability for further biological applications (Figure 1B).

The sharp peak at 230 nm in the UV-Vis spectrum confirms the surface plasmon resonance (SPR) of the SeNPs, as well as their stability. Meanwhile, the peak at 305 nm indicates the loading of metformin onto the SeNP surface, as well as molecular interactions such as hydrogen bonds and electrostatic forces (Figure 1D).

SEM-EDX and elemental mapping results reveal the composition and distribution of the nanoparticles. EDX analysis shows that the sample contains 42.7% C, 41.2% N, 8.5% Se and 7.6% O. The high C:N ratio confirms that metformin has been effectively loaded onto the surface. Mapping images show that the C, N and O elements are homogeneously distributed, while the Se element is present in spots that are consistent with the particle structures. These findings reveal that Met+SeNP is a stable, homogeneous nanocomposite structure, both chemically and morphologically (Figure 1C, E).

### Anthropometric findings

#### Body weight and overweight monitoring

Weight gain was observed in all groups during the first three weeks of letrozole administration, particularly in the PCOS group. During the treatment period (weeks 4-5), weight gain was controlled in the metformin, SeNP, and Met+SeNP nanocomposite groups. The most notable decrease was observed in the Met+SeNP nanocomposite group. The PCOS group experienced significantly higher weight gain than all other groups. (Figure 2A). Treatment with SeNPs alone was found to be more effective than metformin at controlling weight and reducing weight gain (63.10% vs. 43.50%). The Met+SeNP nanocomposite was the most effective option, resulting in the lowest weight gain (39.36%) (see Figure 2B).

At the end of the experiment, the ovarian weights of the PCOS group were significantly higher than those of the healthy group. Meanwhile, in the metformin, SeNP, and Met+SeNP nanocomposite groups, these values were significantly lower (p < 0.05, p < 0.01, and p < 0.01, respectively) (Figure 2C).

### Biochemical findings

#### OGTT measurements, HOMA-IR, and insulin measurements

The OGTT results (Figure 3A-C) showed that the PCOS group developed significant glucose intolerance after 120 minutes. While metformin treatment partially corrected this impairment, SeNP treatment — and Met+SeNP nanocomposite treatment in particular — lowered blood glucose levels to the same level as, or even below, that of the healthy group.

HOMA-IR and insulin levels were significantly higher in the PCOS group than in the healthy group (p < 0.001) (see Figures 3D and 3E). Met+SeNP nanocomposite treatment resulted in significant decreases in these parameters (p < 0.01 and p < 0.001, respectively), with the lowest values obtained in the Met+SeNP group. This group was found to be more effective than the PCOS group and either monotherapy (p < 0.05).

#### Serum hormone findings

LH levels were significantly higher in the PCOS group than in the control group (p < 0.001). Treatment with metformin (p < 0.01) and SeNP (p < 0.001) significantly reduced LH levels, with the strongest effect observed in the Met+SeNP nanocomposite group (p < 0.001) (see Figure 4A). Figure 4B shows that FSH levels were also significantly higher in the PCOS group than in the control group (p < 0.01). Although FSH values decreased in the treatment groups, this decrease was similar to that observed with other treatments, particularly in the Met+SeNP groups, and was not significantly different to that observed in the PCOS group (Figure 4B).

Testosterone levels in the PCOS group were significantly higher than in the healthy control group and the treatment groups (p < 0.001). Metformin and SeNP alone significantly reduced testosterone levels, with the most notable decrease observed in the Met+SeNP nanocomposite group (Figure 4C).

Estrogen levels were significantly lower in the PCOS group than in the control group (p < 0.001). Metformin treatment resulted in an additional decrease in estrogen levels (p < 0.01). An increase was observed with SeNP administration, but it was not significant (p > 0.05). In the Met+ SeNP nanocomposite group, estrogen levels increased significantly compared to the PCOS group (p < 0.01) (Figure 4D).

#### Serum lipid findings

HDL levels were significantly lower (p < 0.05) in the PCOS group, while LDL, total cholesterol and triglyceride levels were significantly higher (p < 0.001). Metformin and SeNP treatments significantly increased HDL levels, with the Met+SeNP nanocomposite providing the most significant increase, approaching the levels observed in the healthy group (p < 0.001). LDL levels decreased partially with metformin treatment, whereas SeNP treatment and, in particular, Met+SeNP nanocomposite treatment showed a stronger reducing effect. Total cholesterol decreased significantly in all treatment groups compared to the PCOS group (p < 0.001) and approached healthy group values in the Met+SeNP group (p > 0.05). Triglyceride levels were high in the PCOS group (p < 0.001), but no significant decrease was observed in the treatment groups (p > 0.05) (Figure 5A-D).

#### Serum hepatic and renal function markers

Serum ALT levels were significantly higher in the PCOS group than in the healthy control group. Both metformin and SeNP monotherapies significantly reduced ALT values, but the greatest reduction was produced by the Met+SeNP nanocomposite, which restored ALT levels to close to those of the healthy group (Figure 6A).

Similarly, serum AST and ALP concentrations were markedly elevated in PCOS rats. While partial improvement was observed following metformin or SeNP administration, treatment with the Met+SeNP nanocomposite resulted in significantly lower AST and ALP levels than in the PCOS group and the groups receiving single treatments (Figures 6B and 6E). Serum urea levels did not differ significantly among the experimental groups, indicating preserved renal function (Figure 6C).

Serum creatinine levels were significantly higher in the PCOS group than in the healthy control group, and were significantly reduced following metformin, SeNP and Met+SeNP treatments. The lowest values were observed in the Met+SeNP group (Figure 6D).

### Histological and immunohistological findings

#### Hematoxylin and eosin staining findings

In the PCOS group, numerous large cystic follicles and primary and secondary follicles surrounded by antral fluid were observed in the cortex. In the Met group, the number of cystic follicles was significantly reduced, with the antral fluid in the centre being replaced by scar-like mesenchymal tissue. The SeNP group exhibited numerous cystic follicles and degenerated primary and secondary follicles, similar to those observed in the PCOS group. In the Met+SeNP nanocomposite group, almost no cystic follicles were evident, resembling the Met group. The few cystic follicles that were present were very small (see Figure 7).

No pathological findings were observed in the muscle tissue (Figure 7). In liver tissue, droplet-shaped lipid accumulation (steatosis) around hepatocytes was evident in the PCOS group. While lipid accumulation was almost absent in the metformin group, steatosis like that observed in the PCOS group was evident in the SeNP group. The Met+SeNP nanocomposite groups had an overall healthy appearance, with rare lipid accumulation detected (see Figure 7).

#### Immunohistochemical staining findings

Quantitative values were generated based on semi-quantitative immunohistochemical scoring and image-based assessment of staining intensity.

Quantitative analysis demonstrated a marked reduction in INSR, IRS1, and IRS2 immunoreactivity in both skeletal muscle (Table 1, Figure 8) and liver tissues (Table 2, Figure 8) of PCOS rats. Metformin and Met+SeNP treatments significantly restored insulin signaling proteins compared with the untreated PCOS group (p<0.001), whereas SeNP monotherapy produced only a mild, statistically non-significant improvement.

## Discussion

SEM images revealed that the Met+SeNPs exhibited spherical or semi-spherical morphology and homogeneous distribution. The average particle size was estimated to be in the range of 150-180 nm. Literature generally reports spherical SeNP structures in the 70-200 nm range for those synthesized with biological or polymeric coatings. Indeed, one study demonstrated the spherical morphology of biogenic SeNPs in the 70-175 nm range using TEM/SEM 32. These findings suggest that the obtained nanoparticles are consistent with those reported in the literature in terms of shape and size. UV-Vis analysis revealed an SPR band specific to SeNPs at 230 nm, which was similar to the 210-250 nm range reported for pure SeNPs 32. The band observed at 305 nm after metformin loading indicated electron transfer associated with the drug. Similarly, the literature reports that new bands specific to the load appear in addition to the carrier nanoparticle band when different drugs (e.g. spirulina) are loaded onto SeNPs 33. These results suggest that metformin loading does not alter the morphology of the nanoparticles. The high C and N ratios observed in EDX analyses confirm that metformin is bound to the surface. Çetin et al. also reported higher C and N ratios in drug-loaded SeNPs than in bare SeNPs 34.

PCOS is an endocrinopathy associated with hormonal imbalances that cause morphological changes in the ovaries. These changes include an increased number of cystic follicles and an enlarged ovarian volume. Both of these features are included in the diagnostic criteria. In our study, we assessed ovarian changes associated with PCOS by measuring weight. Post-experiment measurements revealed significantly increased ovarian weights in all PCOS-induced groups compared to the healthy control group. Similarly, Uhunmwangho et al. found higher ovarian weights in the letrozole-induced PCOS model than in the control group 35. Although ovarian weights increased in the Met+SeNP nanocomposite group, the values obtained were closer to those of the healthy group. The increase observed in the SeNP group was smaller than that in the Met group.

Letrozole disrupts the conversion of testosterone to estrogen by inhibiting the aromatase enzyme. This results in increased total testosterone (tT) levels and decreased estradiol (E2) levels. In our study, tT levels increased and E2 levels decreased in the PCOS group. These results are consistent with those reported by Xu et al. and Mihanfar et al. in letrozole-induced PCOS models 36, 37. Among the treatment groups, the Met+SeNP nanocomposite brought tT levels closer to those of the healthy group, significantly improving E2 levels. Metformin treatment alone reduced tT levels compared to the PCOS group but did not reach the levels seen in the healthy group. These findings support the balancing effect of SeNPs on sex hormones in PCOS, whether taken alone or in combination with metformin 38. When evaluated in terms of gonadotropins, increased levels of both LH and FSH were observed in the PCOS group. These results are consistent with those reported by Kafalı et al. and Younas et al. in letrozole-induced PCOS models 39, 40. The LH/FSH ratio was elevated in the PCOS group, reflecting hyperandrogenism and impaired estrogen levels. In the treatment groups, particularly those receiving Met+SeNP, LH and FSH levels approached those observed in the healthy group. This lowered the LH/FSH ratio, restoring hormone balance to near-normal levels.

Previous studies reported an increase in cystic follicles and a decrease in corpus luteum in a letrozole-induced PCOS model 41, 42. Additionally, Shieh et al. noted that while the granulosa layer thinned in PCOS rats, the theca layer thickness increased 43. Our findings also showed that, in the PCOS group, the granulosa layer had been lost, and the ova were now surrounded by a dense theca layer. In the treatment groups, the number of cystic follicles decreased, and metformin administration significantly improved the outcome. Balasubramanian et al. also demonstrated that metformin treatment reduces the number of cystic follicles and supports corpus luteum formation 42. In our study, SeNP alone had a limited effect, whereas combining it with metformin produced stronger results. An increased number of cystic follicles, a decreased corpus luteum and an altered granulosa-theca layer structure were observed in the PCOS group. Treatment with Met+SeNP normalized both hormone levels and ovarian morphology, bringing them closer to those of the healthy group. In previous studies reported that the application of SeNP alone and in combination with metformin had a positive effect on ovarian morphology 38.

Previous studies have reported beneficial effects of selenium nanoparticles and metformin in experimental PCOS models, including improvements in insulin signaling pathways 38. In the present study, we extend these findings by employing a chemically integrated metformin-selenium nanocomposite and a hormonally driven letrozole-induced PCOS model without dietary manipulation. This design enabled isolation of endocrine-related pathology and clearer mechanistic interpretation of therapeutic responses independent of obesity-related metabolic confounders. The Met+SeNP formulation ensured co-delivery of both agents to ovarian, hepatic, and skeletal muscle tissues, which may account for its superior efficacy compared with monotherapies. Moreover, the selected SeNP dose (0.4 mg/kg) balanced therapeutic benefit with safety, consistent with previous reports demonstrating efficacy at this level 10, 26. This structural integration, as opposed to simple co-administration of two separate agents, may facilitate the concurrent delivery of both therapeutics to target tissues, potentially enhancing their synergistic efficacy.

In our study, weight gain was observed in the other groups alongside the development of PCOS, compared to the healthy group. The highest increase was detected in the PCOS group. Similarly, Rajan and Balaji reported significant weight gain in a letrozole-induced PCOS model 44. Among the treatment groups, the Met+SeNP nanocomposite stood out, with this group showing a weight gain rate similar to that of the healthy group. Previous studies also reported that SeNP and Met+SeNP treatments were effective in controlling weight, with the combination group achieving particularly good results 38. In our study, SeNP and Met+SeNP nanocomposite treatments emerged as effective options for PCOS, reducing weight gain. Impaired glucose metabolism was also observed in the PCOS group alongside weight gain.

The absence of significant differences between SeNP and Met+SeNP in body weight and OGTT parameters may be related to the inherent metabolic effects of SeNPs and the limited treatment period. Nevertheless, Met+SeNP generally produced greater improvements in HOMA-IR, serum insulin levels, and insulin signaling proteins than SeNP alone, with the most pronounced effects observed in insulin signaling markers. These findings suggest that the nanocomposite may provide additional molecular benefits beyond those detected by body weight and OGTT measurements.

In our study, the results of the OGTT showed a significant increase in the PCOS group compared to the healthy group. This suggests impaired glucose tolerance and IR. While metformin maintained blood glucose levels close to those of the healthy group, the SeNP and Met+SeNP treatment groups further reduced values by increasing glucose utilisation. The insulin levels of the PCOS group were also found to be significantly higher than those of the healthy group. This finding is consistent with the results of El-Bahaie et al 45. Abdulmalek and Balbaa also reported that the Met+SeNP nanocomposite kept fasting insulin levels lower than those of the healthy group 10. HOMA-IR measurements confirmed the success of our experimental model. Although HOMA-IR was high in the PCOS group, the Met+SeNP nanocomposite group, which reduced both fasting plasma glucose (FPG) and insulin levels, produced the lowest HOMA-IR value.

Insulin resistance associated with PCOS primarily results in decreased insulin receptor response in muscle and liver tissue, followed by decreased response in other tissues. The phosphorylation of IRS-1 and IRS-2, which play a role in the intracellular signaling of the insulin receptor, mediates insulin's metabolic events. Insulin resistance occurs in conditions such as PCOS, diabetes, obesity and metabolic syndrome and is characterized by the desensitization of insulin receptors and downstream signaling molecules. Failure to treat insulin resistance, which underlies the etiology of many diseases, can lead to serious health problems, primarily cardiovascular complications. Studies have shown that insulin resistance associated with PCOS causes impairment of INR, IRS-1 and IRS-2, at a cellular level, primarily in muscle and liver tissue, but also in other organs 23, 37,46,47.

In a previous study, we demonstrated that the levels of the INR, total IRS-1 and total IRS-2 were reduced in liver tissue in a letrozole-induced PCOS model when examined using immunohistochemistry. In groups treated with sacubitril/valsartan, however, these levels were observed to improve 23. In our study, INR, IRS-1 and IRS-2 expression was reduced in the livers of the PCOS-induced groups, and metformin treatment improved these protein levels. Treatment with SeNP alone did not have a significant effect, whereas Met-SeNP nanocomposite treatment produced the expected results. These findings are consistent with biochemical results.

Muscle tissue accounts for 70-80% of glucose uptake in the body and is a critical target tissue in terms of insulin sensitivity. When insulin resistance develops in PCOS, the muscles are unable to absorb sufficient glucose, which increases the risk of hyperglycemia and type 2 diabetes. In our rat model, IR impairment was observed in the muscle tissue of the PCOS-induced groups. In a previous study, we found that the expression of the INR, total IRS-1 and IRS-2 in muscle tissue was reduced in the PCOS model compared to the healthy group. However, this expression improved in groups treated with sacubitril/valsartan 23.

In our study, INR and total IRS-1 and IRS-2 protein levels decreased in the PCOS-induced groups. Metformin treatment increased INR, total IRS-1 and IRS-2 protein levels in muscle tissue. In contrast, Corbould et al. reported normal protein levels but reduced functional activity; disruption of the PI3K pathway may be responsible for this 48. Similarly, Abd El-Tawab, Tammam et al. reported decreased expression of the INR and the IRS-1 and IRS-2 in the muscles of diabetic patients. They also reported increased expression of these genes when metformin or telmisartan was administered 49. No significant effect of SeNP treatment was observed on INR, IRS-1 or IRS-2. It is thought that the effect of combination therapy is due to metformin.

Another indicator of metabolic disorders is the lipid profile. Women with PCOS are at high risk of hyperlipidemia, which is linked to impaired glucose tolerance and insulin resistance 1, 3. In our study, HDL levels decreased and LDL, total cholesterol and triglyceride levels increased in the PCOS group. These findings are consistent with those reported in the literature on letrozole-induced PCOS models 50, 51. The lipid profiles of the treatment groups improved, with the Met+SeNP nanocomposite bringing LDL and total cholesterol levels closest to those of the healthy group, while also significantly increasing HDL. Although metformin alone had a positive effect on HDL levels, they remained slightly lower with combination therapy. However, Met+SeNP was the most effective treatment for LDL and total cholesterol. Triglyceride levels did not change significantly in the treatment groups, although a slight decrease was observed in the metformin and Met+SeNPs groups. These results support the synergistic effect of the Met+SeNP nanocomposite in improving lipid metabolism in PCOS 10, 38.

In this study, letrozole-induced PCOS was associated with significant elevations in serum ALT, AST, and ALP levels, indicating hepatic metabolic stress and hepatocellular injury. These alterations are consistent with previous reports showing that endocrine disruption and insulin resistance in PCOS contribute to hepatic steatosis and low-grade inflammation 38, 52.

Metformin treatment partially corrected these abnormalities, in agreement with its known hepatoprotective and insulin-sensitizing properties 16, 17. Selenium nanoparticles alone produced modest improvement, whereas the metformin-functionalized selenium nanocomposite exerted the strongest effect in reducing ALT, AST, and ALP levels. This superior response may reflect the combined actions of selenium-mediated antioxidant defense and metformin-driven metabolic regulation. Selenium nanoparticles have been shown to attenuate hepatic injury through suppression of oxidative stress and inflammatory pathways 53, while metformin activates AMPK signaling and improves hepatic insulin sensitivity.

Serum urea levels remained unchanged among groups, indicating preserved renal function in the hormonally induced PCOS model. Although serum creatinine was mildly elevated in the PCOS group, its normalization following Met+SeNP treatment suggests that the nanocomposite did not exert nephrotoxic effects. This finding is in line with toxicity studies showing that selenium nanoparticle doses below 0.5 mg/kg are generally well tolerated in female rats 26.

Taken together, these results indicate that Met+SeNP therapy not only improves reproductive and metabolic disturbances in PCOS but also provides hepatic protection without compromising renal safety, thereby strengthening the translational relevance of this nanotherapeutic approach.

In conclusion, metformin-functionalized selenium nanoparticles significantly ameliorated endocrine and metabolic disturbances in a letrozole-induced PCOS rat model. The nanocomposite improved glucose tolerance, insulin resistance, lipid abnormalities, and ovarian histopathology while restoring insulin receptor signaling in liver and skeletal muscle tissues.

### Limitations

Ovarian insulin signaling molecules (IRS-1, PI3K, Akt) and insulin tolerance testing (ITT) were not included in the present study because the primary aim was to evaluate the therapeutic efficacy of the newly synthesized metformin-functionalized selenium nanocomposite on systemic metabolic parameters and insulin signaling proteins in major insulin-responsive tissues (liver and skeletal muscle). Nevertheless, evaluation of ovarian insulin signaling and downstream mediators represents an important direction for future studies.

Insulin sensitivity was assessed using OGTT, fasting insulin levels, and HOMA-IR, which are widely used indicators of insulin resistance in experimental PCOS models. However, the absence of ITT may be considered a limitation of the study.

Furthermore, no *in vitro* validation using PCOS-relevant cell models such as granulosa or theca cells was performed, which represents an additional limitation of the present study.

A further limitation of this study is the absence of a co-administration group receiving free metformin plus free SeNPs. Consequently, the present data demonstrates the superiority of Met+SeNP over each monotherapy individually rather than over a conventional combination treatment, and this limitation should be taken into account when interpreting the translational advantages claimed for the integrated nanoformulation. Future studies incorporating a free Met + free SeNP co-administration arm are needed to conclusively establish the added value of structural integration over simple combination therapy.

Another limitation is that pharmacokinetic, encapsulation efficiency, formulation stability, and drug-release studies were not performed for the present Met+SeNP formulation. Although similar selenium nanoparticle systems developed by our group have demonstrated these properties in previous studies 21, 34, these characteristics should not be assumed for the current formulation without direct experimental confirmation.

## Figures and Tables

**Figure 1:**
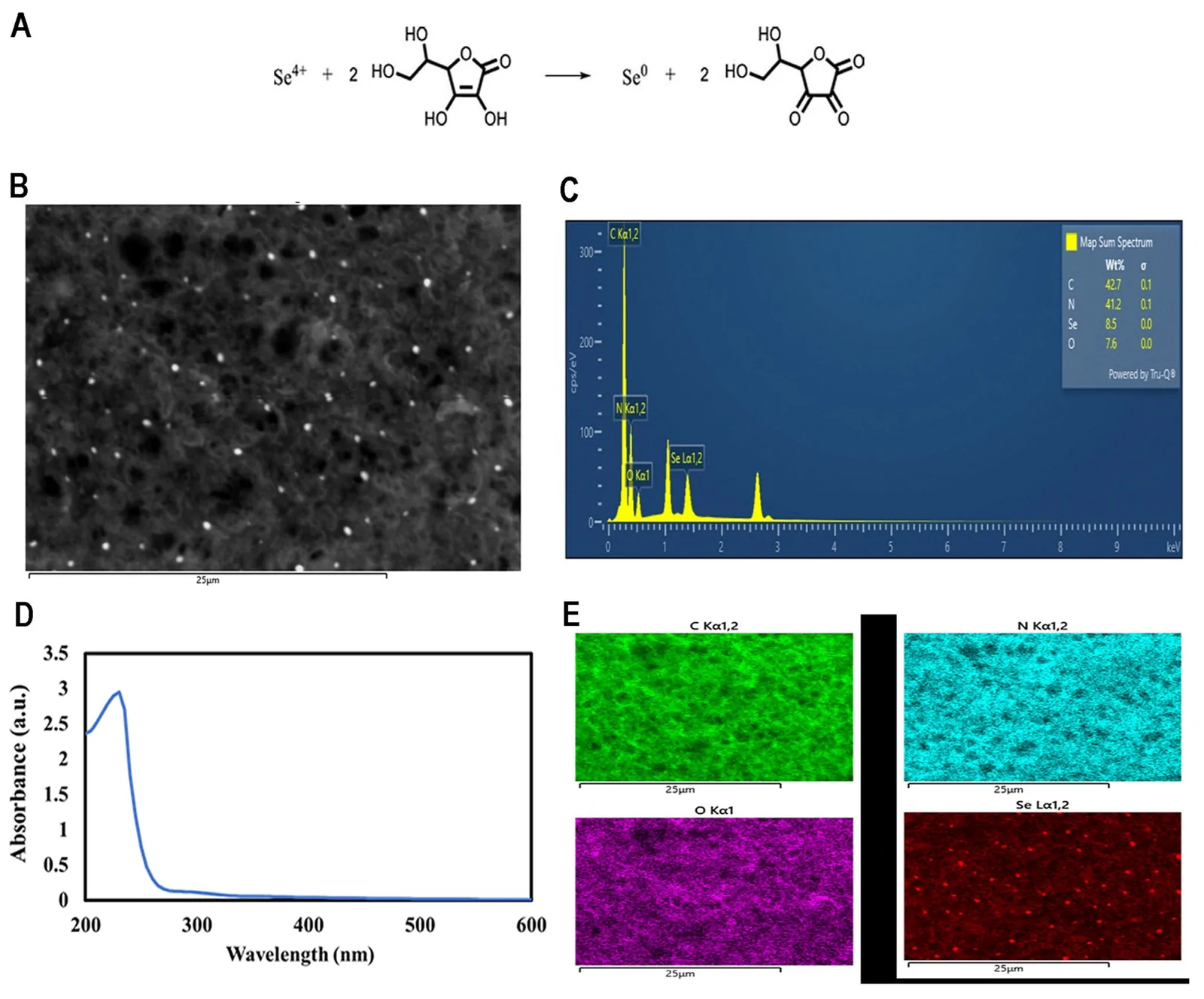
Mechanism of SeNP Synthesis (A). SEM image of metformin-loaded SeNPs (B). SEM EDX of metformin-loaded SeNPs (C). UV-Vis Spectrum of metformin-loaded SeNPs. nm: Nanometer, a.u: Absorbance Unit (D). E: Elemental mapping of metformin-loaded SeNPs (E).

**Figure 2:**
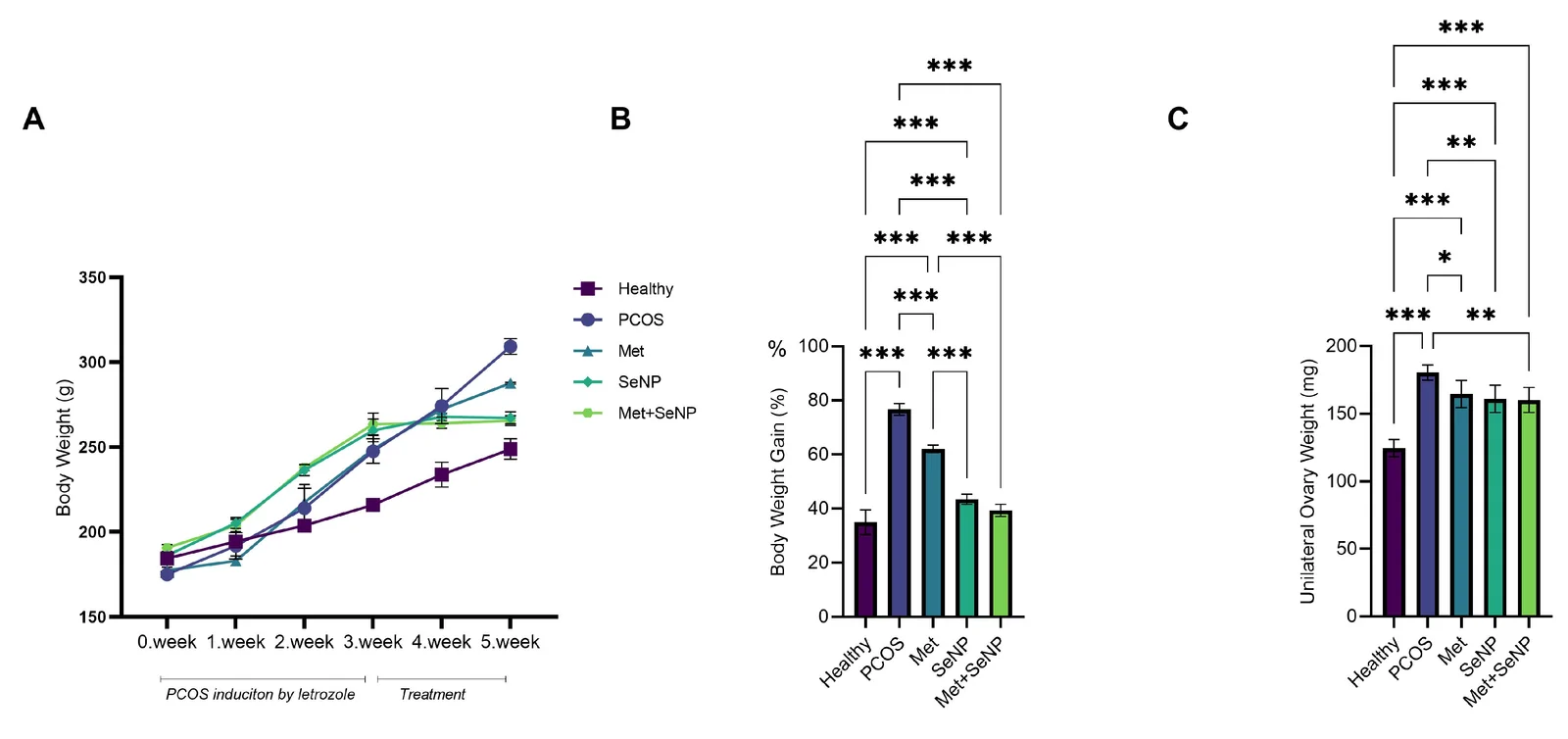
Metabolic parameters in the letrozole-induced PCOS rat model across experimental groups. (A) Body weight changes during the experimental period. (B) Weight gain at the end of the experiment. (C) Single ovarian weight. Data are presented as mean ± SD. Statistical comparisons between groups were performed, and significance levels are indicated as follows: *p<0.05; **p<0.01; ***p<0.001; ns: not significant.

**Figure 3:**
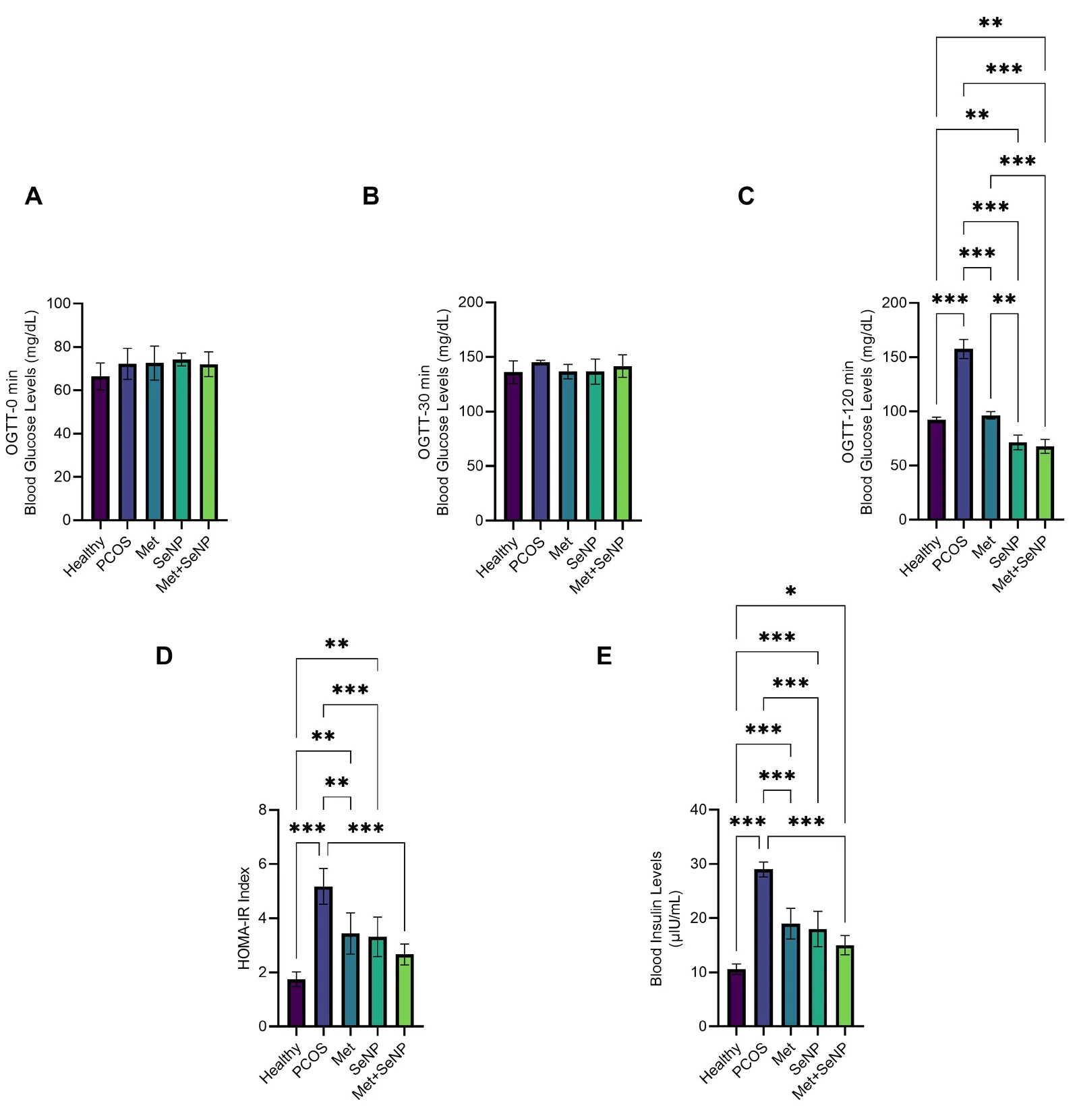
Serum metabolic parameters in the letrozole-induced PCOS rat model across experimental groups. (A-C) Oral glucose tolerance test (OGTT) results: plasma glucose levels at 0 min (A), 30 min (B), and 120 min (C). (D) HOMA-IR values. (E) Serum insulin levels. Data are presented as mean ± SD. Statistical comparisons between groups were performed, and significance levels are indicated as follows: *p<0.05; **p<0.01; ***p<0.001; ns: not significant.

**Figure 4:**
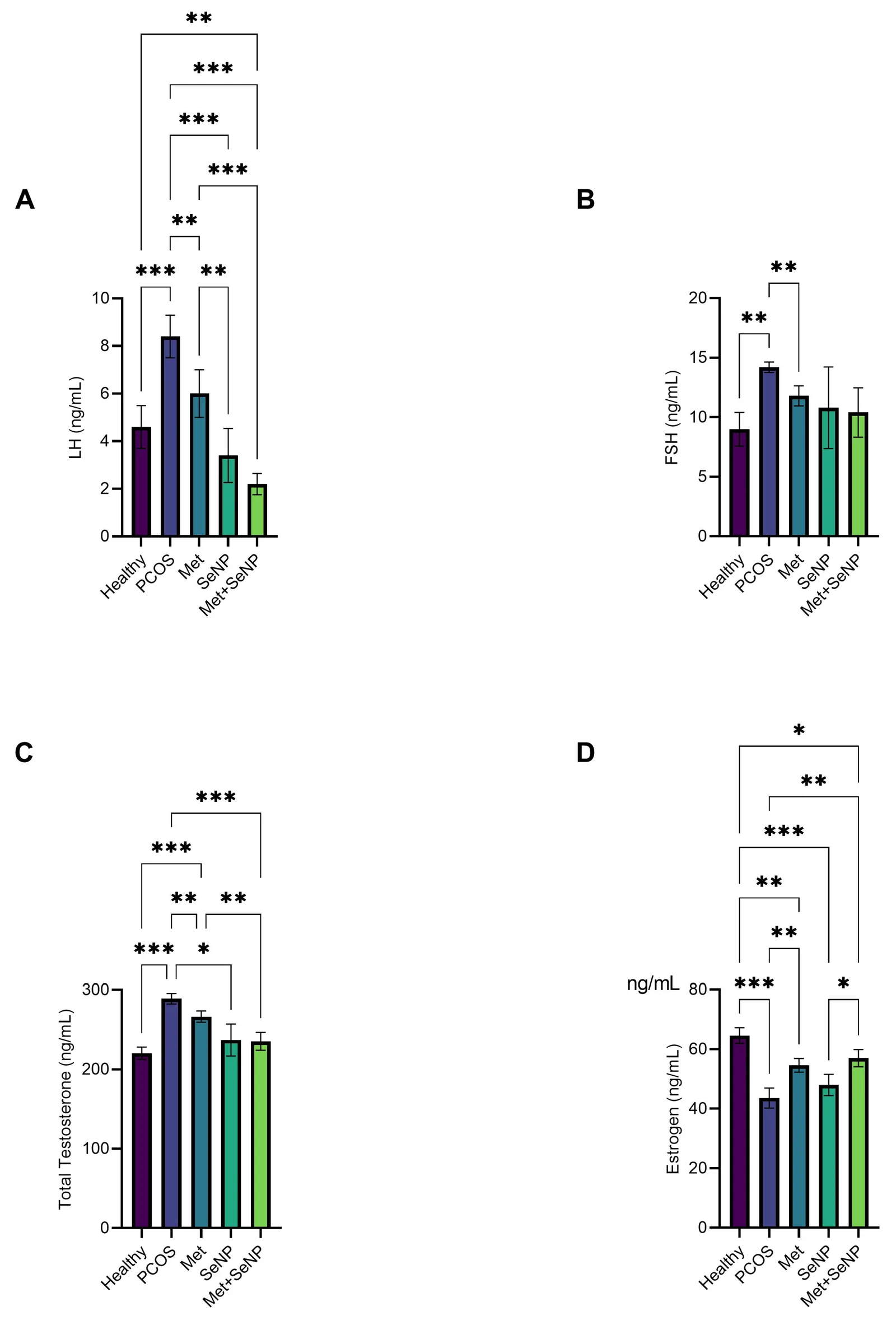
Serum reproductive hormone levels in the letrozole-induced PCOS rat model. (A) Luteinizing hormone (LH) (B) Follicle stimulating hormone (FSH). (C) Total testosterone. (D) Estradiol (E2). Data are presented as mean ± SD. Statistical analyses were performed between groups, and significance levels are indicated as follows: *p<0.05; **p<0.01; ***p<0.001.

**Figure 5:**
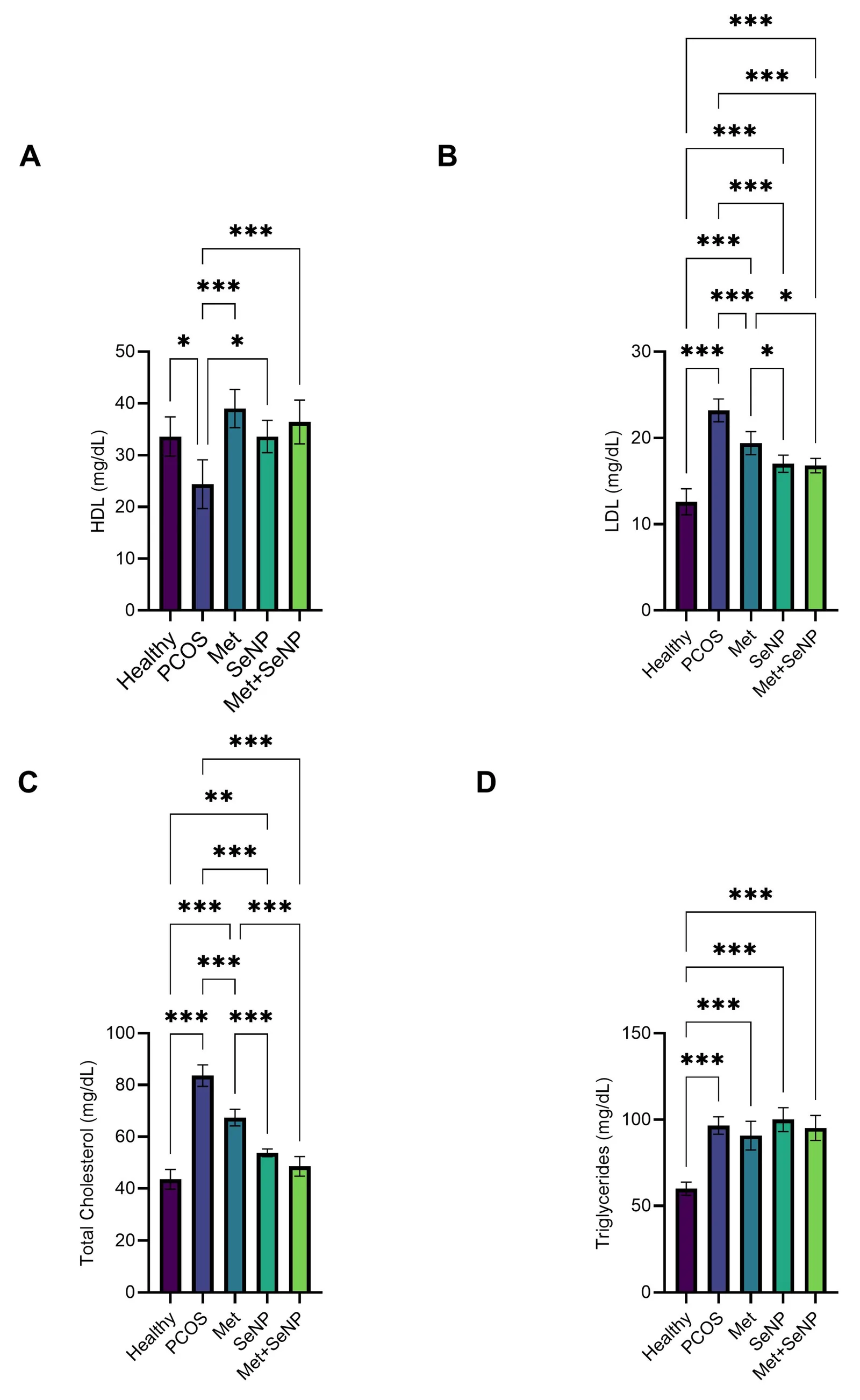
Serum lipid profiles in the letrozole-induced PCOS rat model. (A) High Density Lipoprotein-Cholesterol (HDL). (B) Low Density Lipoprotein-Cholesterol (LDL). (C) Total cholesterol (D) Triglycerides. Data are presented as mean ± SD. Statistical analyses were performed between groups, and significance levels are indicated as follows: *p<0.05; **p<0.01; ***p<0.001.

**Figure 6:**
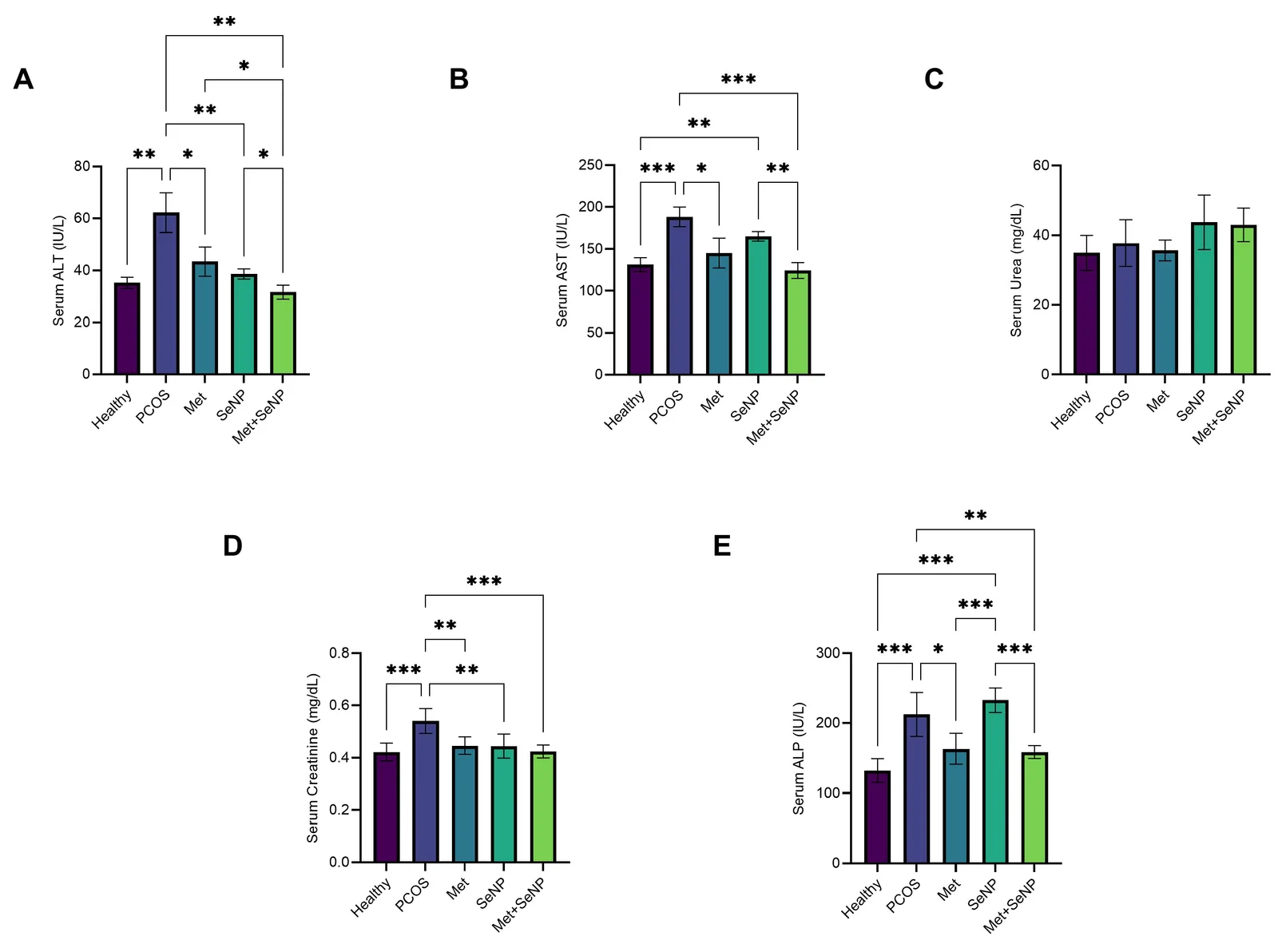
Serum hepatic and renal biochemical parameters in the letrozole-induced PCOS rat model. Serum levels of alanine aminotransferase (ALT) (A), aspartate aminotransferase (AST) (B), urea (C), creatinine (D), and alkaline phosphatase (ALP) (E) in healthy control, PCOS, metformin, SeNP, and Met+SeNP-treated groups. Data are presented as mean ± SD. Statistical analyses were performed between groups, and significance levels are indicated as follows: *p<0.05; **p<0.01; ***p<0.001.

**Figure 7:**
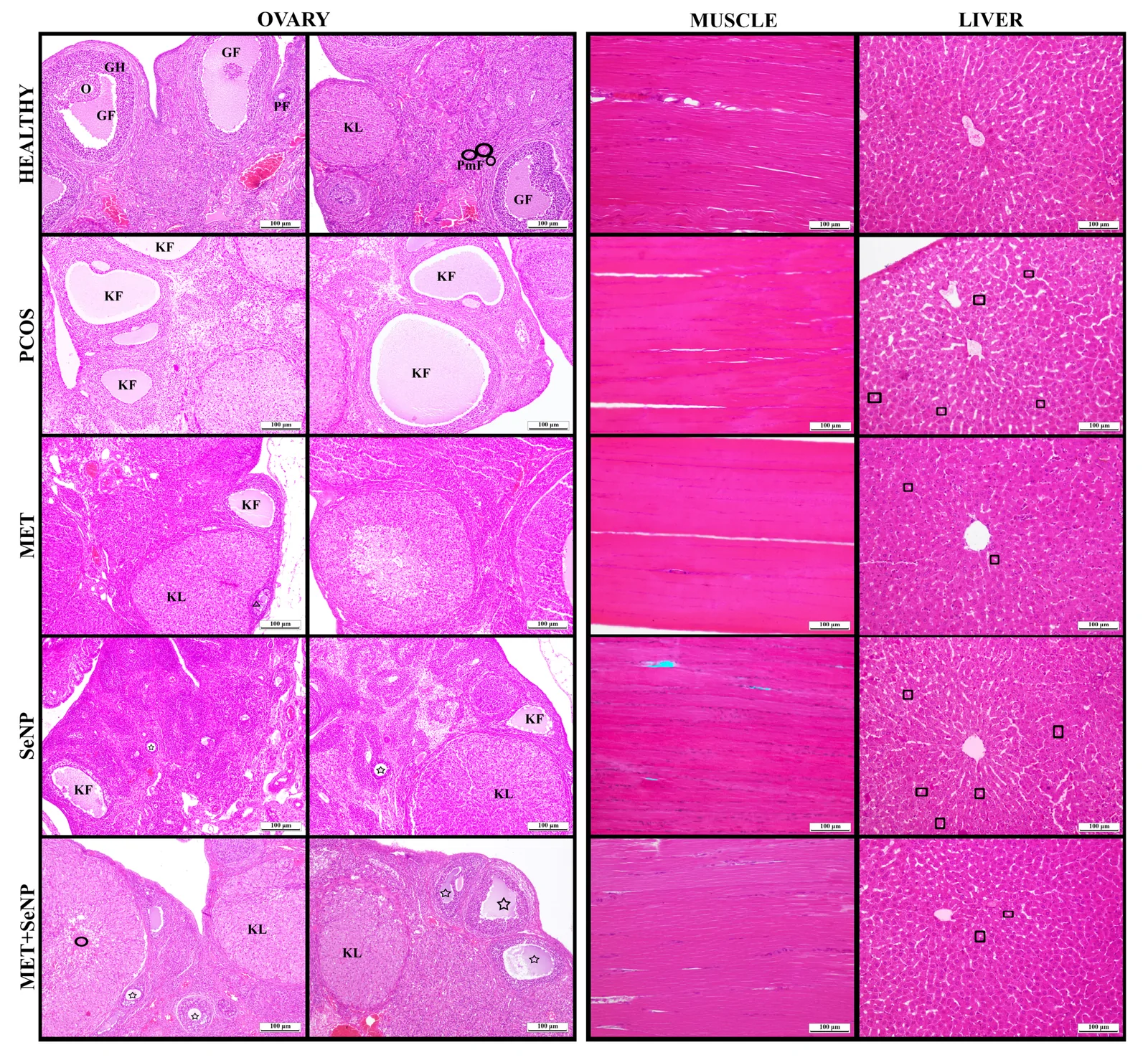
Hematoxylin and Eosin (H&E) staining findings in ovarian, muscle, and liver tissues. (PmF: Primordial Follicle, PF: Primer Follicle, KL: Corpus Luteum, GH: Granulosa Cell, O: Oocyte, KF: Cystic Follicle, GF: Graafian Follicle, Asterisk: Newly developing follicles, Circle: Mesenchymal tissue, Square: Steatosis lipid accumulations, Scale bars = 100 μm.)

**Figure 8:**
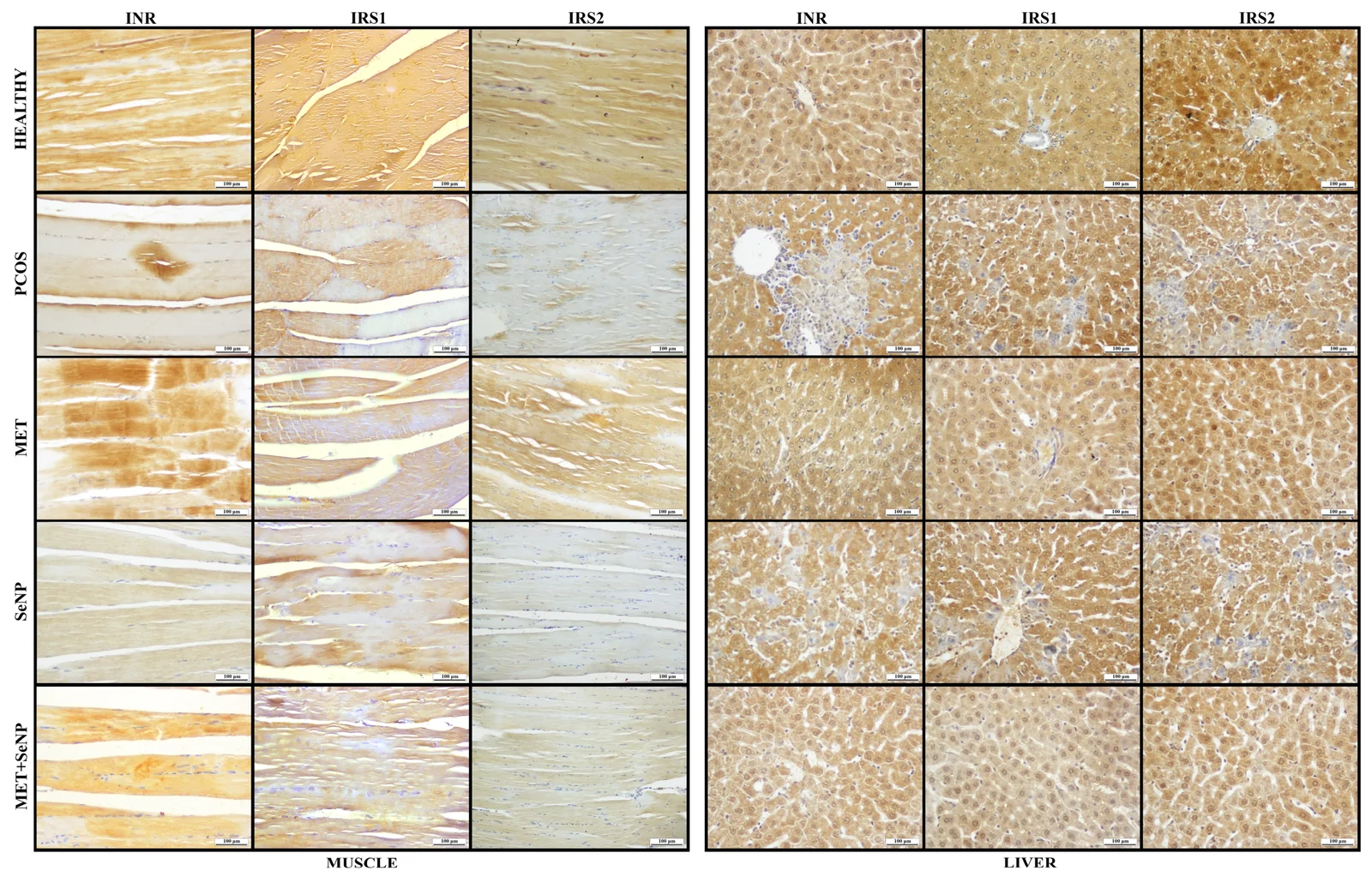
Immunohistochemical staining of insulin receptor (INR), insulin receptor substrate-1 (IRS-1), and insulin receptor substrate-2 (IRS-2) in muscle and liver tissues. (Positive immunoreactivity is indicated in brown (DAB staining), while nuclei are counterstained with hematoxylin blue. Scale bars = 100 μm).

**Table 1:** Quantitative analysis of skeletal muscle insulin signaling proteins (% positive area)

Group	INR (%)	IRS1 (%)	IRS2 (%)
Healthy	70.8 ± 4.6	74.5 ± 4.9	72.6 ± 4.8
PCOS	19.7 ± 3.3 ^***^	37.1 ± 4.0 ^***^	18.4 ± 3.1 ^***^
Met	47.5 ± 4.2 ^***^ ^ ###^	68.3 ± 4.5 ^###^	45.8 ± 4.0 ^***^ ^ ###^
SeNP	22.1 ± 3.5 ^***^ ^ +++^	39.5 ± 4.3 ^***^ ^ +++^	21.2 ± 3.4 ^***^ ^ +++^
Met+SeNP	46.4 ± 4.4 ^***^ ^ ###^ ^ &&&^	70.7 ± 4.6 ^###^ ^ &&&^	24.7 ± 3.7 ^***^ ^ +++^

INR: Insulin receptor, IRS1: Insulin receptor Substrate-1, IRS2: Insulin receptor Substrate-2. Values are presented as mean ± SD. *** p<0.001 vs Healthy; ### p<0.001 vs PCOS; +++ p<0.001 vs Met; &&& p<0.001 vs SeNP.

**Table 2:** Quantitative analysis of hepatic insulin signaling proteins (% positive area)

Group	INR (%)	IRS1 (%)	IRS2 (%)
Healthy	85.4 ± 4.8	89.1 ± 5.0	92.1 ± 4.9
PCOS	21.3 ± 3.7 ^***^	38.4 ± 4.1 ^***^	19.8 ± 3.2 ^***^
Metformin	82.1 ± 4.6 ^###^	86.2 ± 4.8 ^###^	88.1 ± 4.4 ^###^
SeNP	24.6 ± 4.0 ^***^ ^ +++^	36.9 ± 4.5 ^***^ ^ +++^	22.7 ± 3.5 ^***^ ^ +++^
Met+SeNP	84.1 ± 4.5 ^###^ ^ &&&^	87.9 ± 4.7 ^###^ ^ &&&^	89.2 ± 4.6 ^###^ ^ &&&^

INR: Insulin receptor, IRS1: Insulin receptor Substrate-1, IRS2: Insulin receptor Substrate-2. Values are presented as mean ± SD. *** p<0.001 vs Healthy; ### p<0.001 vs PCOS; +++ p<0.001 vs Met; &&& p<0.001 vs SeNP.
